# Analysis of Surface Current by Quantum Tunneling Effect of Thin Film Transistors with Topological Insulators

**DOI:** 10.1038/s41598-020-66499-4

**Published:** 2020-06-11

**Authors:** Teresa Oh

**Affiliations:** Department of Semiconductor Engineering, Cheongju University, Cheongju, 28503 Republic of Korea

**Keywords:** Two-dimensional materials, Single photons and quantum effects

## Abstract

Dirac insulator and Weyl conductors have different semiconductor structures. A Dirac insulator is a SiOC insulated thin film, and a Weyl conductor consists of transistors with different semiconductor structures combining channels with SiOC insulated films. The transfer characteristics of transistors were investigated in this study. The difference between Dirac insulators and Weyl conductors is the same as the difference between transistors without channels and transistors with channels. Transistors without channels exhibit bidirectional transmission characteristics due to the spin currents of the Dirac insulators. By contrast, transistors with channels display unidirectional transmission characteristics consistent with the movement of the charges in the channels. This unidirectional transmission characteristic results in an existence of the threshold voltage and leakage current.

## Introduction

Electromechanical energy is common to all physical systems. Electric fields are highly influential in the physical world at the macroscale, wheresa magnetic fields are relatively influential at the microscale. The quantum Hall effect is a conductive phenomenon that occurs in response to magnetic energy and is associated with a magnetic resistance. Similarly, superconductivity is a kind of conductivity that arises due to magnetic energy. Thus, in the absence of magnetic fields, the quantum Hall effect is meaningless. However, there are reports of surface currents flowing even without magnetic fields in 2-dimensional (2D) semiconductor materials; this phenomenon is called the quantum anomalous Hall effect. In a bidirectional transistor, current flows even without the application of a magnetic field because potential barriers are formed by the gate insulators, and such a potential barrier itself is a form of magnetic energy. Therefore, the quantum anomalous Hall effect is a general quantum Hall effect that arises with magnetic resistance in the structure of a 2D PN-junction semiconductor. Dirac-isolators and Weyl conductors both have magnetic resistance^1^ properties; in particularly the current decreases as the temperature increases, corresponding to a negative resistance. A Dirac insulator has only a magnetic resistance, whereas since a Weyl conductor has an electric charge corresponding to impurities, it has both a magnetic resistance, which is negative and an ohmic resistance, which is positive^2^.

Recently, research results indicating a tunneling phenomenon in magnetic resistance characteristics have drawn considerable attention from researchers^3,4^. However, reports of transistors based on magnetic resistance are hard to find. At present, several ways are available to improve the performance of transistors by means of tunneling and trapping effects, which tend to be so similar as to be unable to be clearly distinguished. This is an effect of using multiple different carriers. Because defects and impurities are introduced into the thin films, leakage current problems are common^5,6^, and there is a limitation on how small and thin such devices can be. In other words, the use of high concentrations of impurities and combinations presents a fundamental problem of certain physical limitations that cannot be overcome in the attempt to miniaturize semiconductors. Transistors traditionally have either N-type or P-type electrical characteristics, and single-directional transmission characteristics, depending on their impurities. Recently, however, there have been reports of transistors with bidirectional transmission characteristics^7^. The author has previously published a paper reporting the results of a study on tunneling phenomena in transistors, in which bidirectional transfer characteristics are observed when a gate insulation films with good insulation properties are used because of the reduction of the polarity^6^. In ZTO thin film transistors^8^, IGZO thin film transistors^9^, SnO_2_ thin film transistors^10^ and organic thin film transistors (OTTTs)^11^ a factor of 10 increase in mobility over a typical OTFT has been reported. The space-charge limited currents in the depleted layer, which are chiral currents, are the same as surface currents flowing in the insulator. Low-k SiOC thin films have excellent amorphous properties due to nuclear reactions driven by the bimolecular nucleophilic substitution reaction (S_*N*_2) mechanism^6^, which results in good surface current flow. Surface currents are the principal currents passing through phase isolators and exhibit a magnetic resistance characteristic. The surface current of a phase isolator has been reported to be capable of creating quantum tunneling phenomena due to quantum confinement effects^12–14^. Sodha^15^ has reported that the higher the potential barrier is in a dielectric with a very low dielectric rate, the more likely quantum tunneling is to occur, and Wiegmann^16^ has interpreted the one-dimensional Fermi theory as a plane model.

## Chiral Currents and Surface Currents

**Figure 1:**
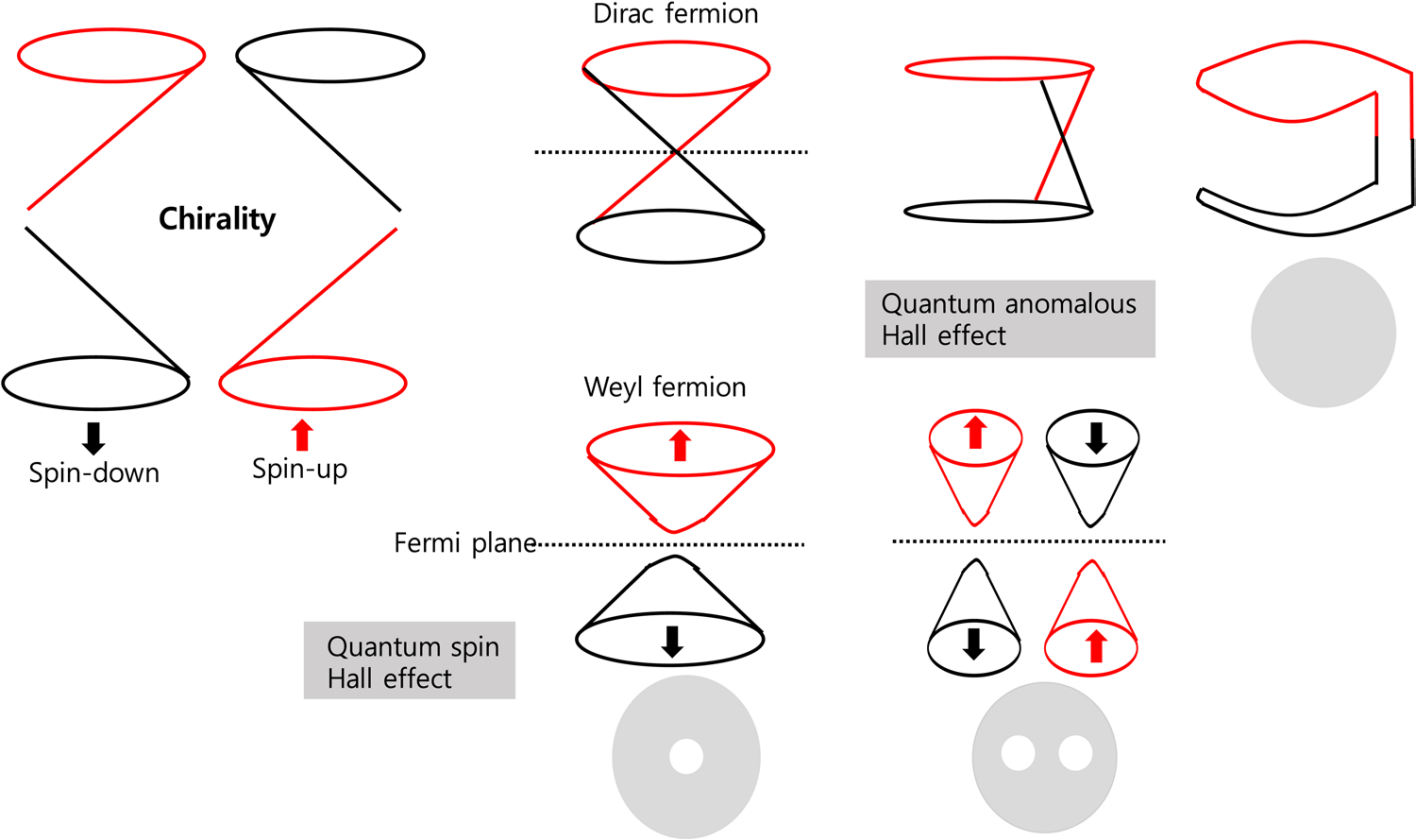
A phase isolator with no channels due to the chirality of the surface current and the quantum anomalous Hall effect and a Weyl conductor with channels.

**Figure 2:**
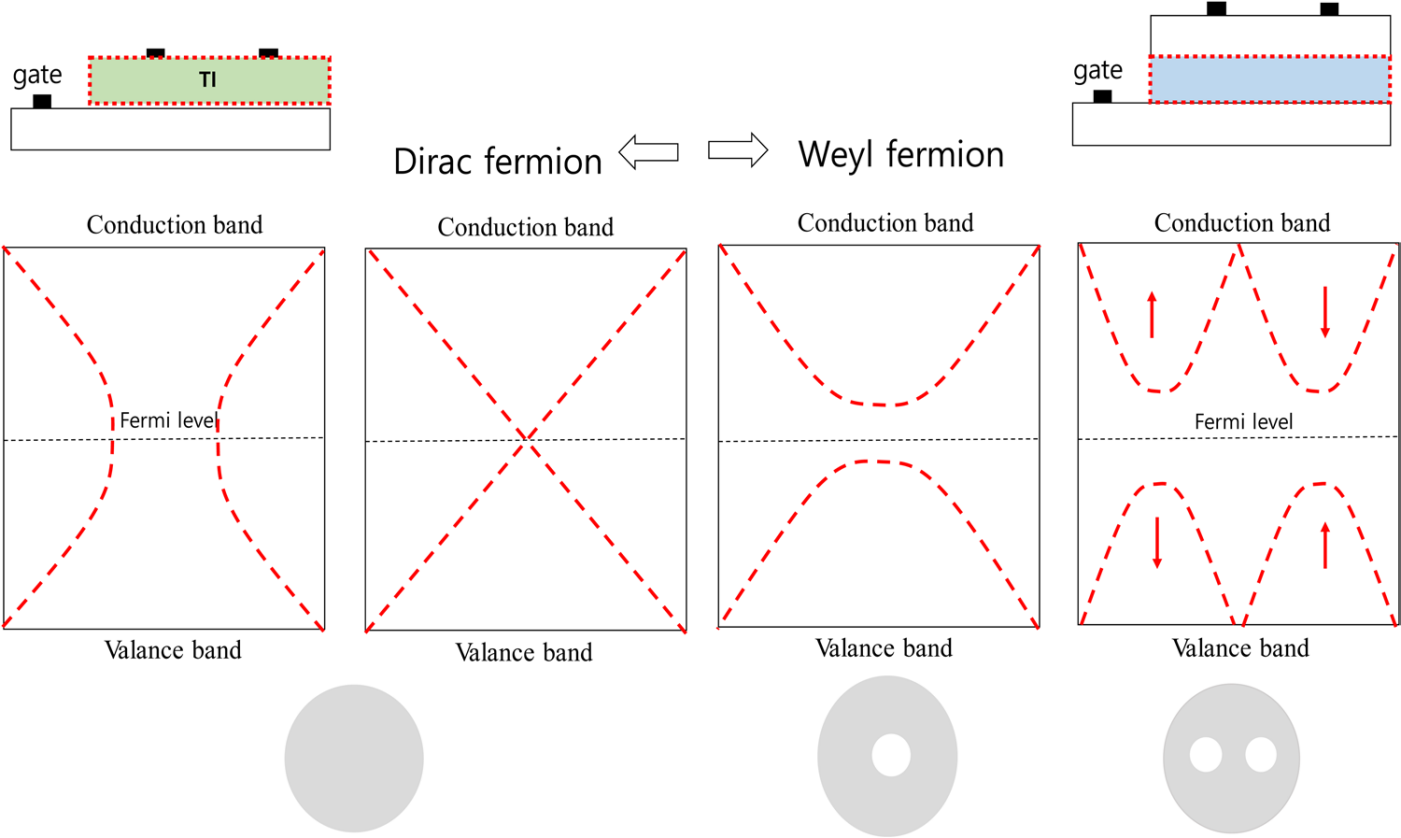
Fermion effects in a unidirectional transistor due to channel conductivity and in a bidirectrironal transistor in which a surface current flows.

A band structure following the Dirac function is a linear energy-momentum graph, in where the electromagnetic energy and conduction band meet at a single point and a band gap does not exist. The point at which the electromagnetic energy meets the conduction band is defined as the Dirac point, and the corresponding band structure is referred to as the Dirac cone structure. Similarly, the band structure of a phase isolator is also a Dirac cone structure in which Dirac points exist^17,18^. A nonmassive fermion is a particle that follows the Fermi-Dirac function and is either a Dirac fermion or Weyl fermion depending on the behavior of the spin current. Dirac fermions compose the spin current flow in a phase isolator and carry spins in both directions. By contrast, a Weyl fermion carries a spin in only one direction. A Weyl metal is a normal semiconductors state with excitation resulting from low magnetic energy inside the conductor and having the properties of Weyl fermions. Thus, the physical phenomena in a Dirac insulator occurring at high magnetic energies are determined by the quantum properties of Dirac fermions. The topology describing a phase isolator is related to the conductivity given by the states of the first Brillouin zone, whereas the topology describing Dirac insulator and Weyl metal are related to the conductivity on the Fermi surface^19,20^. The quantum Hall effect describes a number of ways in which electrons can exist in a two-dimensional system. When there is a strong external magnetic field acting in a rectangular region, the motion of an electron inside this region is circular. On the other hand, an electron on an edge of the rectangle exhibits continuous movement in one direction. In other words, there exists a concept of conductive edges, which we call quantum Hall edges. This concept can serve as a basis for expanding either the quantum Hall effect or the quantum spin Hall effect. The idea is that in a traditional two-dimensional system, we can implement a conductive edge without the need for a strong external magnetic field. This presents various possibilities^21,22^. The quantum anamolous spin Hall effect refers to the formation of a quantum Hall state as a result of forces induced by the inherent motion properties of an electron itself instead of the motion caused by a strong external magnetic field. Spin-orbit coupling is an important concept that supports the forces induced by such electron motion. Spin-orbit coupling refers to the cause in which an electron’s unique spin and its orbital degree of freedom are coupled. Such coupling gives rise to a force induced by the spin of the electron itself acting within an amorphous structure without the need for an external magnetic. This induced force moves the electron along the edge as described above. Because the electron may be either spin-up or spin-down, with opposite spins leading to motion in opposite directions, it is possible to move in both directions rather than being restricted to a single direction^23,24^. In conclusion, both the quantum Hall effect and the quantum spin Hall effect result in metallic behavior at the edges of magnetic field regions, but not inside them. Now, let us describe the principle of a two-dimensional phase structure with insulation. From a band structural point of view, there is a certain energy level on the surface, It can be inferred that the ground level is located within the band gaps; hence, the ground level can be said to be insulated^25,26^. The current produced by a massless fermion is a chiral current and is symmetrical. Such a chiral current cannot be carried by mass-bearing fermions. A topological spin current (phase quantization), corresponding to Dirac fermions is associated with the Dirac node in the Brillouin domain, whereas the topology of a Weyl metal is related to the Weyl node on the Fermi surface and to the chirality or surface state, of the Weyl fermions. In classical mechanics, a chiral current is formed only under a magnetic field. Specifically, the phenomenon in which a chiral current occurs even with a magnetic field is called the Hall effect. If an electric field is applied parallel to the magnetic field, all states will shift slightly due to the energy dispersion in the momentum space of the massless Dirac fermions. This is the chiral anomaly effect that occurs in the case of Weyl fermions, for which chirality is produced /destroyed differently than it is for other particles. As the total number of particles decreases, magnetic resistance decreases and magnetic energy increases. In addition, a variety of electromagnetic phenomena, such as the chiral magnetic effect of a Weyl metal manifest, as a result of this chiral anomaly^27^. As seen in Fig. 1, the Dirac fermion flows continuously and continuously through the Dirac node, because of one electron with up and down spins, in the absence of magnetic fields. This phenomenon is called the quantum spin Hall effect, and is topologically similar to bread with no holes. Even if the magnetic field is not large, the Wely fermion is susceptible to the effect of this weak magnetic field because of spread spins of many electrons; as a result, the conduction and the valence bands are separated from the Fermi plane and the Fermi level falls within the forbidden zone. Topologically, this situation can be described as bread with holes. Since there is no Dirac node, the spin current is weakened and magnetic fields are formed by the surface current as shown in Fig. 2. Therefore, phase isolators can be divided into Dirac insulators with spin current only and Weyl conductors with both surface and spin currents.

**Figure 3:**
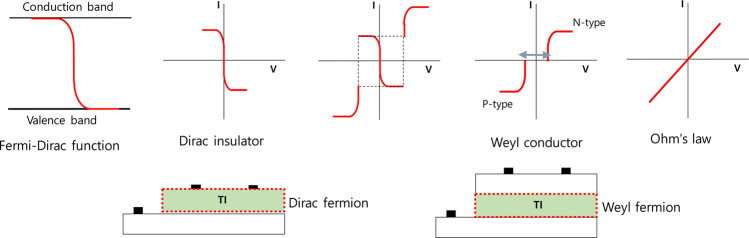
Relationship between the electrical characteristics of transistors and phase isolators.

Figure 3 illustrates the correlation between the energy distributions of the fermions and the operating principles of the transistors. Transistors with phase isolation, in which spin currents flow will have bidirectional transmission characteristics. On the other hand, Weyl fermions have conductivity properties characteristics of trapping in the presence of a surface current or impurities, separating the conduction band from the valence band. Thus, Weyl conductors with channel/insulated-film structures exhibit unidirectional transmission characteristics, which vary depending on the channel characteristics and their topology can be characterized as a doughnut, i.e., there is a topological hole. This hole corresponds to the need to find a new center point to compensate for the lack of stability as the center of the phase disappears^28,29^. As illustrated in Fig. 3, transistors can be generally divided into N-type and P-type transistors, depending on the impurities in the channels and they are directional when they operate above a certain threshold voltage. Therefore, the voltage-current transfer characteristics are unidirectional. By contrast, phase-isolator transistors do not have a channel layer but instead are operated on the basis of the spin current of the gate-insulating film and exhibit bidirectional transmission characteristics. These bidirectional transmission characteristics resemble the Fermi-Dirac distribution function^30^.

Just as the usual transistor transfer characteristics described by semiconductor theory require a threshold voltage, the phenomenon of a Weyl node in a Weyl conductor is similar to the formation of the band structure of a transistor. However, bidirectional transistors are a phenomenon that occurs only in phase-isolators. These transistors exhibit bidirectional transmission characteristics, and they are characterized by a negative magnetic resistance due to the potential barriers formed by the phase isolators.

In this study, phase-isolator transistors and Weyl-conductor transistors were manufactured to investigate the transfer characteristics of each type of transistor.

## Experimental Procedures

A SiOC as a gate insulator was prepared on a Si wafer with an oxygen flow rate of 20 sccm using a magnetron sputtering system operating at 250 RF power for 10 min. The source target was a SiOC target fabricated by Nano Technology, Inc., Korea. The as deposited SiOC films were annealed at various temperaturers under vacuum and ambient condition. The channel material used to make the transistors was IGZO, which was deposited via the magnetron sputtering method with argon gases at 30 sccm. IGZO/SiOC transistors with channels and SiOC transistors without channels were manufactured to investigate their electrical characteristics. Schematic diagrams of the transistors are presented in Fig. 3. The electrical characteristics of the device were measured using a semiconductor parameter analyzer (HP4155A).

## Results and Discussions

**Figure 4:**
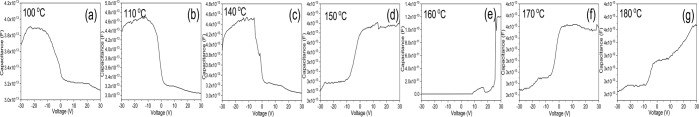
Capacitances of the SiOC thin films subjected to different heat treatment temperatures; (**a**) 100 °C, (**b**) 110 °C, (**c**) 140 °C, (**d**) 150 °C, (**e**) 160 °C, (**f)** 170 °C, (**g**) 180 °C.

Figure 4 shows the capacitances of SiOC thin films subjected to different heat treatment temperatures. A drastic change occurrs between 140 °C and 150 °C, with the semiconductor type switching from P to N. Although the films treated at low temperatures showed characteristics of P-type semiconductors, they transformed to exhibit N-type semiconductor characteristics as the temperature increased.

**Figure 5:**
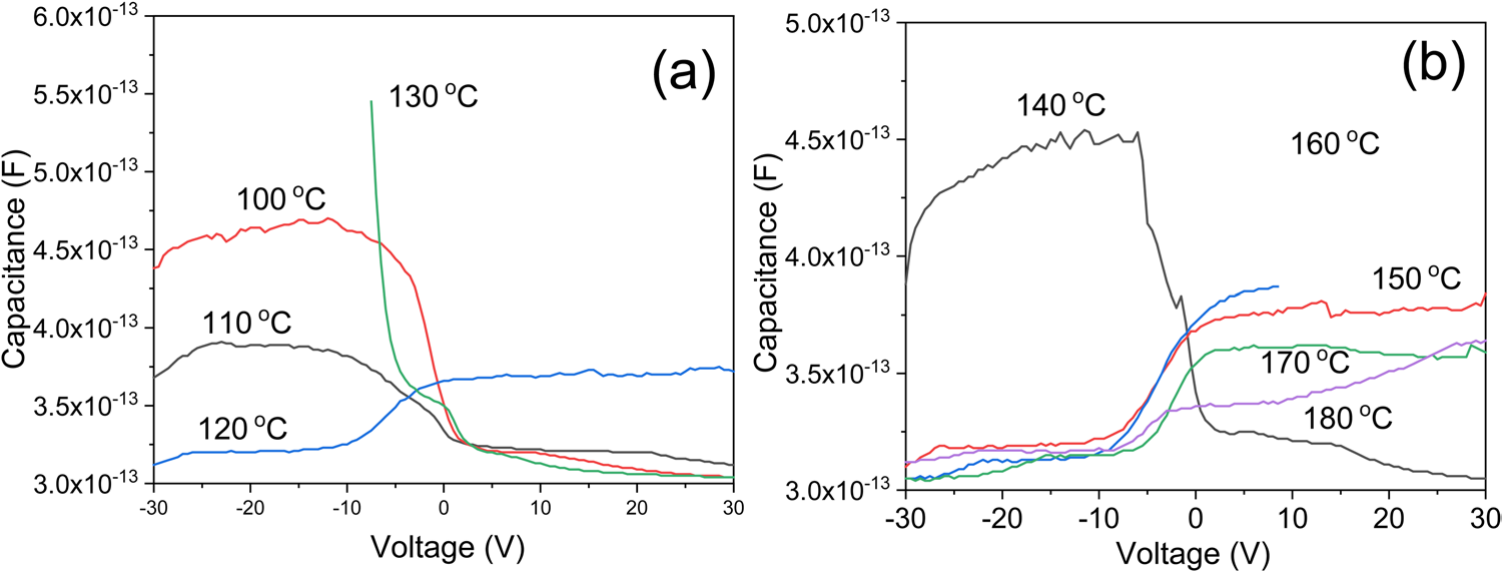
Comparison of relative capaacitance values of SiOC thin films subjected to various annealing temperatures; (**a**) films with P-type properties, (**b**) films with N-type properties.

Figure 5 compares the relative capacitance values. As shown in Fig. 5(a), the capacitance decreased as the temperature increased for the films with P-type semiconductor characteristics, and the capacitance value then abruptly increased in the temperature range, where the change to N-type characteristics occurred from 130 °C to 160 °C. Subsequently, Fig. 5(b) shows that the capacitance again decreased as the temperature increased. The phenomenon of decreasing current with increasing temperature is characteristic of magnetoresistance, and is also a typical characteristic of the quantum spin Hall effect.

**Figure 6:**
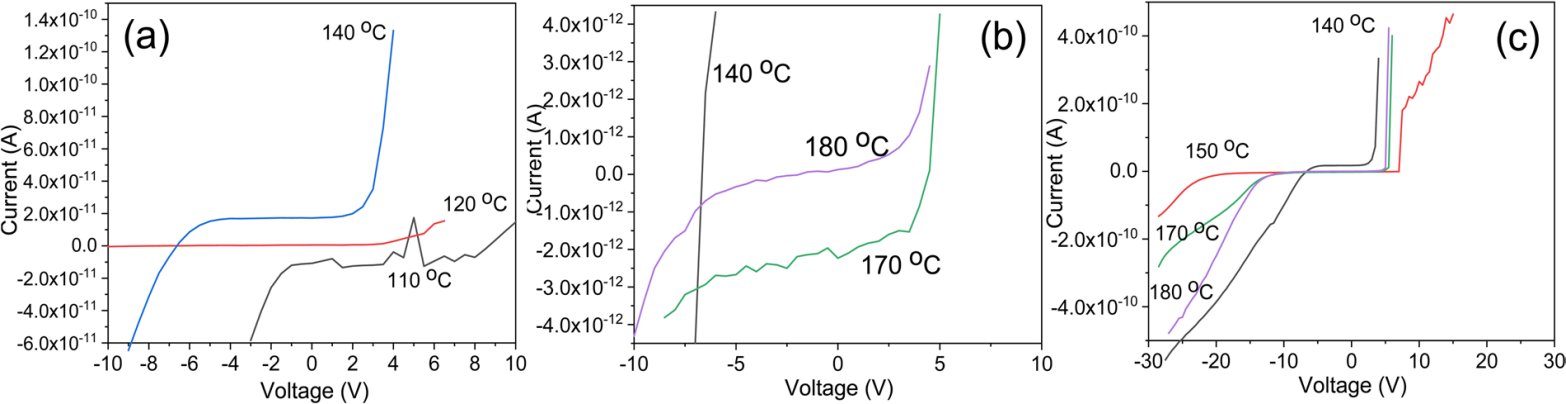
Current-voltage characteristics of SiOC thin films treated at different temperatures; (**a**) P-type films, (**b**) N-type films, and (**c)** films corresponding to the transistion from P-type to N- type.

Figure 6 illustrates the current-voltage characteristics of the SiOC thin films treated a different temperatures. The highest current flows was achieved through treatment at 140 °C. For higher temperatures the current decreases with increasing temperature. Decreasing current with increasing temperature is a characteristic of magnetoresistance, specifically a negative resistance, which is one of the characteristics of a material with a quantum-Hall effect. SiOC thin films form potential barriers to act as insulating films, and the better the insulation characteristics are, the higher the potential barrier for phase insulation. Such a potential barrier has a magnetoresistance characteristics and, if an electric field is applied, a potential barrier proportional to the applied field with form in the opposite direction, which will have a magnetic energy effect. Thus, even without a magnetic field being applied, the quantum spin Hall effect can arise, causing the current to decrease as the temperature increases, Even in the absence of a magnetic field, a surface current will be generated on the insulator allowing the transistor to operate with bidirectional transmission characteristics.

**Figure 7:**
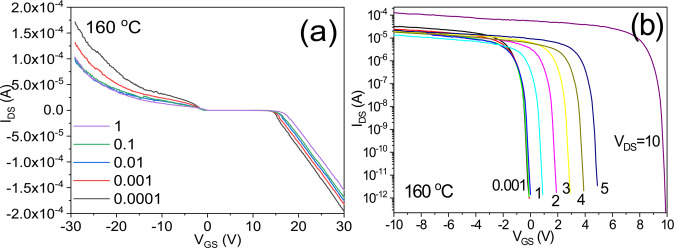
I_DS_-V_GS_ and I_DS_-V_DS_ transfer characteristics of a transistor treated at 160 °C, (**a**) on a linear scale, and (**b**) on a logarithmic scale.

Figure 7 shows the electrical characteristics of a transistor that is operated using only a gate insulating film, without a channel structure. Specifically, the I_DS_-V_GS_ transfer characteristics of a SiOC thin film transistor that was heat treated at 160 °C are shown. The results illustrate the bidirectional transfer characteristics with a positive I_DS_ current flowing when V_GS_ is negative and a negative I_DS_ current flowing when V_GS_ is positive. The lower the voltage V_DS_ is the better the I_DS_-V_GS_ characteristics. Figure 7(b) shows the I_DS_-V_GS_ characteristics on a logarithmic scale. As the voltage V_DS_ decreases, the performance increases. These characteristics differ from the typical behavior of a unidirectional transistor, for which the performance tends to improve as the voltage V_DS_ increases.

**Figure 8:**
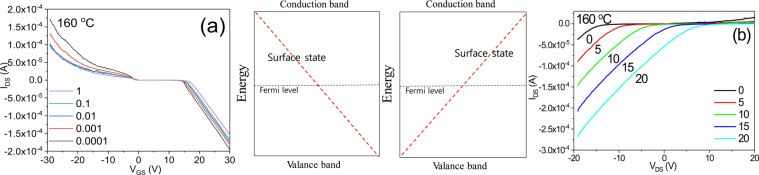
Characteristics of a bidirectional transistor operated on the basis of the surface current of a phase isolator; (**a**) I_DS_-V_GS_ and (**b**) I_DS_-V_DS_.

The electrical I_DS_-V_GS_ and I_DS_-V_DS_ characteristics are further illustrated in Fig. 8. The characteristics of the surface current of a phase isolator are similar to the characteristics of the energy dispersion in the momentum space of massless Dirac fermions.

**Figure 9:**
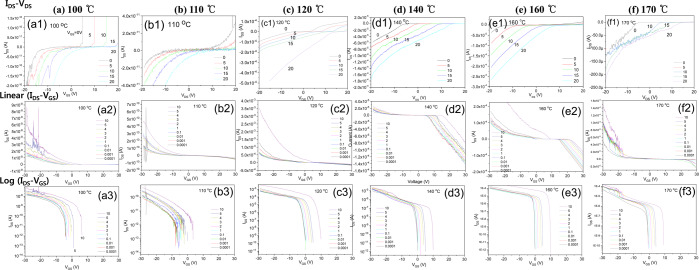
Transfer characteristics of I_DS_-V_GS_ and I_DS_-V_DS_ on transitsors with various annealing temperatures; (**a**) 100 °C, (**b**) 110 °C, (**c**) 120 °C, (**d)** 140 °C, (**e**) 160 °C, **(f**) 170 °C.

Figure 9 shows the electrical I_DS_-V_GS_ and I_DS_-V_DS_ characteristics of transistors subjected to different heat treatment temperature. The top row shows I_DS_-V_DS_ plots, the middle row shows I_DS_-V_GS_ plots, and the bottom row shows the I_DS_-V_GS_ plots on a logarithmic scale. The electrical properties of thin films heat treated at 160 °C are the best.

**Figure 10:**
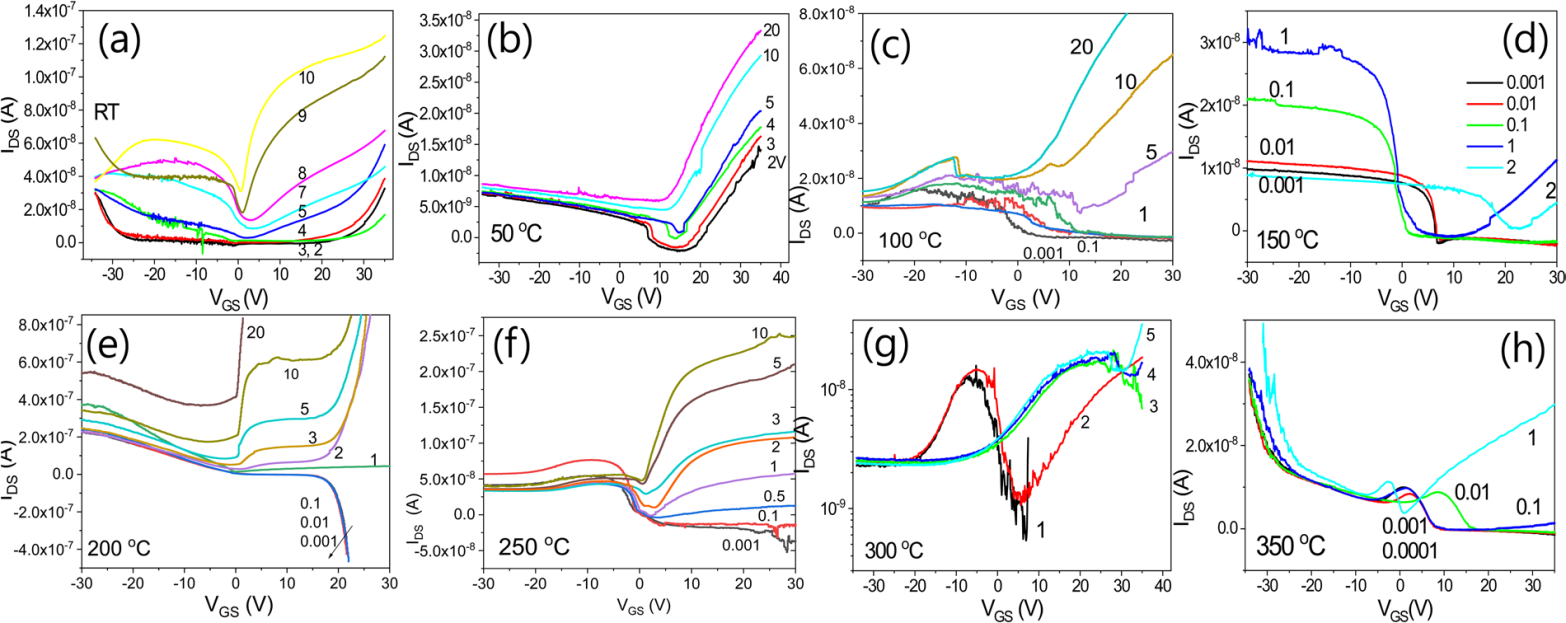
I_DS_-V_GS_ transfer characteristics of IGZO/SiOC transistors treated at various annealing temperatures.

Figure 10 illustrates the I_DS_-V_GS_ transfer characteristics for transistors with IGZO/SiOC structures. The I_DS_-V_GS_ transfer characteristics were investigated for 0.001 V ≤ V_DS_ ≤ 20 V. The results for V_DS_ ≤ 1 V indicate bidirectional transmission characteristics, with positive I_DS_ at V_GS_ < 0 V and negative I_DS_ at V_GS_ > 0 V. However, the results for V_DS_ > 1 V shows that as the voltage V_DS_ increases, a shift in the threshold voltage occurs, resulting in a positive I_DS_, only for for V_GS_ > 0 V. For heat treatment temperatures of 200 °C and higher, the bidirectional transmissi°n characteristicsweaken with increasing temperature and the unidirectional transmission characteristics improve as the shift in the threshold voltage decreases in size. As the heat treatment temperature increases, the generation of new charges increases, showing that the strength of the channel effect is increasing and the transistors begin to exhibit unidirectional transmission characteristics. When heat treated a SiOC material forms a depletion zone in which charges recombine causing the amount of free charge to decrease and increasing the barrier potential. This barrier potential behaves as magnetic energy; the barrier potential is the largest at the temperature at which the amount of free charge is the lowest, at which point the characteristics of the phase isolator begin to dominate. To determine the effect of heat treatment on the barrier potentials of SiOC films, the I_DS_-V_GS_ transfer characteristics corresponding to different heat treatment temperatures were compared at V_DS_ = 1 V.

**Figure 11:**
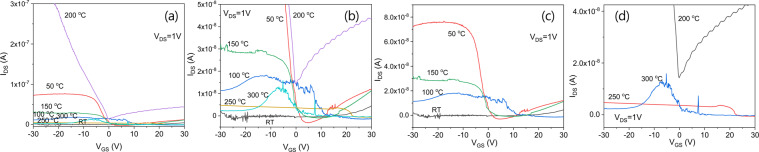
I_DS_-V_GS_ transfer characteristics of IGZO/SiOC transistors treated at various annealing temperatures under the condition of V_DS_ = 1 V.

All transistors showed bidirectional transmission characteristics at V_DS_ = 1 V, and the current I_DS_ in the transistors prepared using SiOC thin films that had been treated at 200 °C was the highest. As the temperature increased, I_DS_ decreased. As shown in Fig. 11(d), I_DS_ showed a rapid drop at V_GS_ = 0 V only in the case of heat treatment at 200 °C. The phase of I_DS_ also changed with the change in the polarity of the voltage V_GS_. This phenomenon is attributed to the characteristics of the phase isolators, which allows a surface current to flow without a magnetic field due to the quantum anomalous Hall effect. This surface current is characterized by very high mobility due to the quantum effect. This is because the potential barrier presented by the SiOC insulating film is very large, resulting in quantum tunneling in the phase insulator. This type of Weyl conductor can be seen to exhibit severe instability due to the threshold voltage shift, even if bidirectional transimission characteristics are present in transistors containing channels. It can also be seen that even for a bidirectional conductor as in the case of 200 °C heat treatment no threshold voltage shift is evident when the gate insulating layer is a phase insulator. Thus the phase insulator ensures the stability of the transistor.

**Figure 12:**
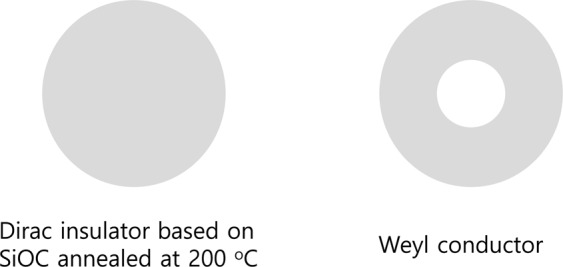
Weyl conductor and Dirac insulator based on different SiOC films.

Figure 12 shows that very good transistor transfers characteristics can be obtained from SiOC films heat treated at 200 °C and that such transistors have the characteristics of phase isolators exhibiting different properties from those of other transistors. Transistors of the Weyl conductor type have lower transmission properties than those of the phase isolator type.

**Figure 13:**
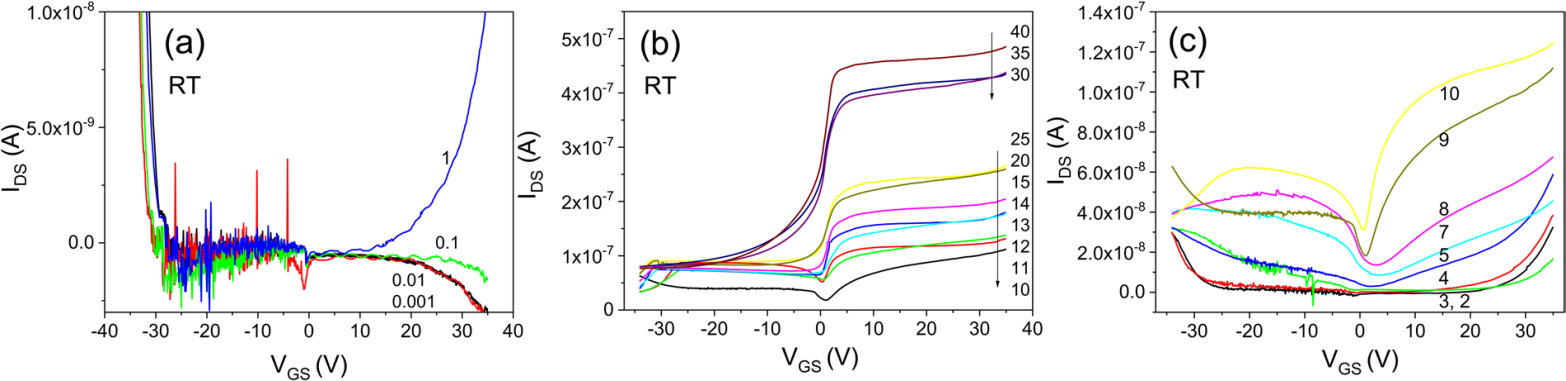
Transfer characteristics of an IGZO/SiOC thin film transistor with as-deposited SiOC as the gate insulator; (**a**) bipolar properties at V_DS_ < 1 V, (**b**) unipoar properties at V_DS_ ≥ 10 V and (**c**) unipolar properties at V_DS_ > 1 V.

Figure 13 shows the transfer characteristics of an IGZO/SiOC thin film transistor with non-heat treated SiOC as the gate insulator. Bidirectional transmission characteristics are evident in the range of V_DS_ < 1 V but the bipolar properties are not good. As the voltage increases to V_DS_ > 1 V unidirectional transmission characteristics appear and the unipolar characteristics are relatively superior.

**Figure 14:**
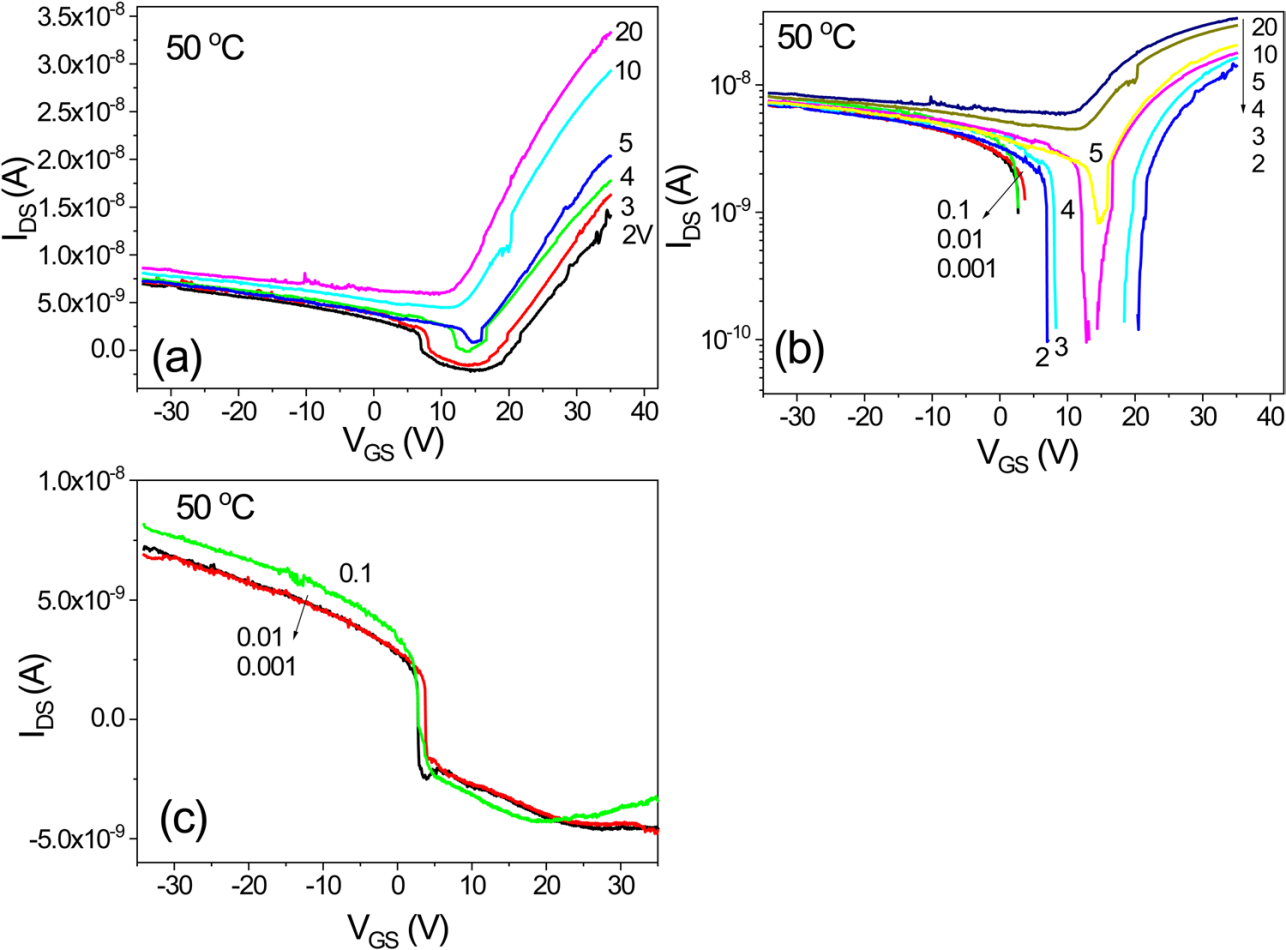
Transfer characteristics of an IGZO/SiOC thin film transistor with SiOC heat treated at 50 °C as the gate insulator; (**a**) electrical properties at V_DS_ ≥ 2 V, (**b**) same as (**a**) on a logarithmic scale and (**c**) electrical properties at V_DS_ < 1 V.

Figure 14 shows the transfer characteristics of an IGZO/SiOC thin film transistor with SiOC heat treated at 50 °C as the gate insulator. At low voltage is low at V_DS_ < 1 V, bidirectional characteristics are observed, and as the behavior becomes unidirectioonal at V_DS_ ≥ 2 V, the threshold voltage shifts to ensure stability as V_DS_ increases.

**Figure 15:**
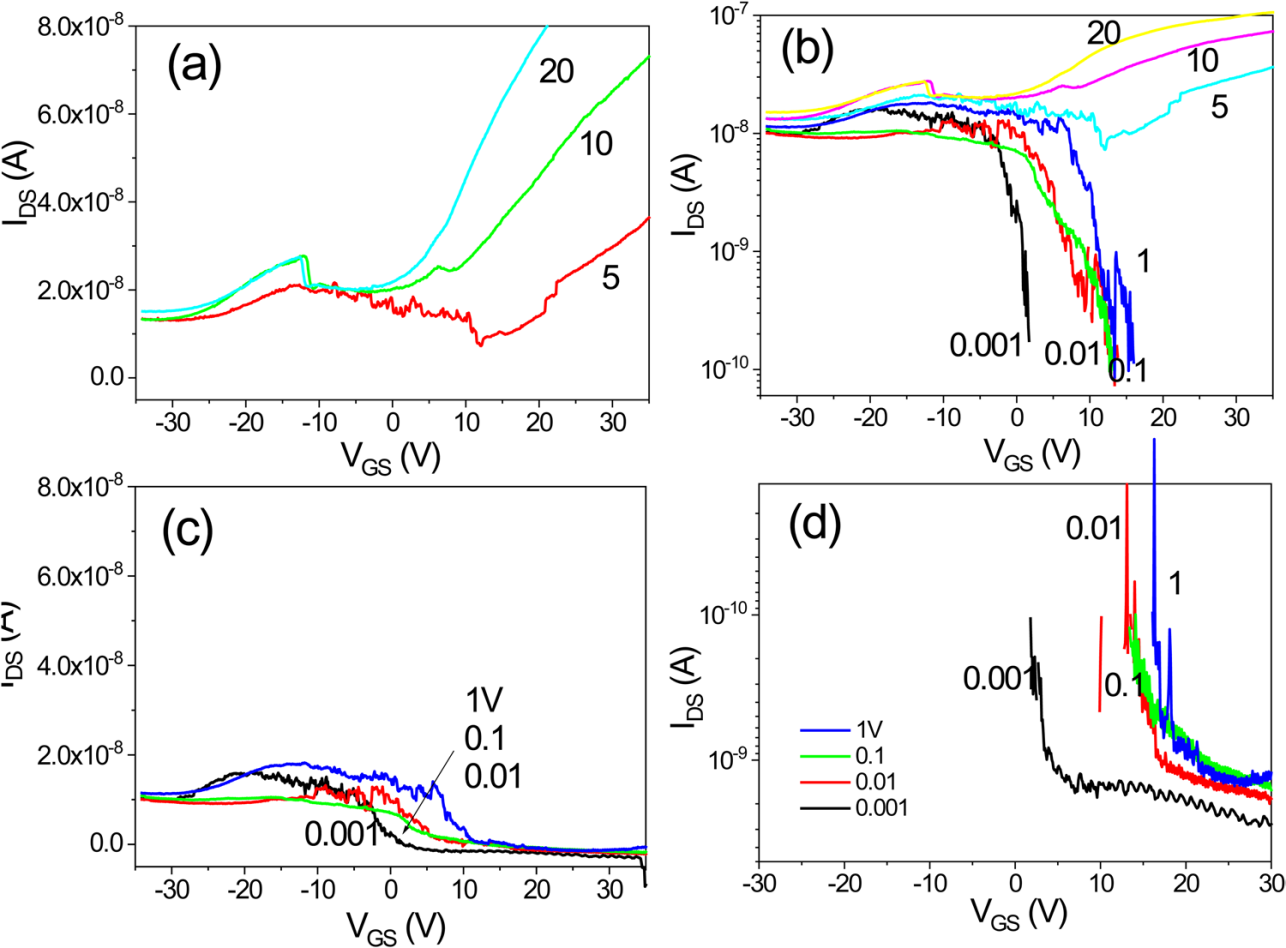
Transfer characteristics of an IGZO/SiOC thin film transistor with SiOC heat treated at 100 °C as the gate insulator; (**a**) electrical properties at V_DS_ ≥ 5 V, (**b**) positive current results on a logarithmic scale, (**c**) electrical properties at V_DS_ < 1 V and (**d**) negative current results on a logarithmic scale.

Figure 15 shows the transfer characteristics of an IGZO/SiOC thin film transistor with SiOC heat treated at 100 °C at the gate insulator. Both bidirectional and unidirectional transmission characteristics are observed, but the electrical characteristics are not improved.

**Figure 16:**
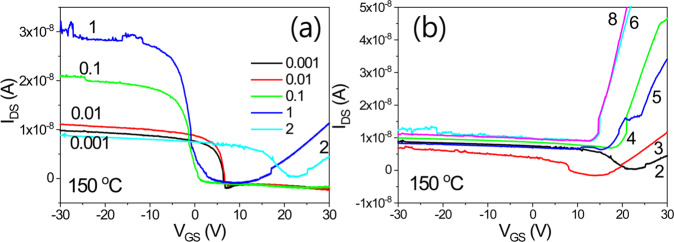
Transfer characteristics of an IGZO/SiOC thin film transistor with heat treated SiOC heat treated at 150 °C as the gate insulator, (**a**) electrical properties at V_DS_ ≥ 2 V and (**b**) electrical properties at V_DS_ < 2 V.

Figure 16 shows the transfer characteristics of an IGZO/SiOC thin film transistor with SiOC heat treated at 150 °C as the gate insulator. The transmission properties mainly show negative charge characteristics. Unidirectional and bidirectional transmission behaviors are both observed but the electrical characteristics are not excellent.

**Figure 17:**
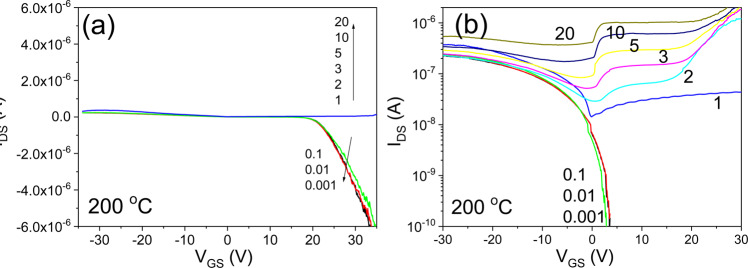
Transfer characteristics of an IGZO/SiOC thin film transistor with SiOC heat treated at 200 °C as the gate insulator, (**a**) on a linear scale, (**b**) on a logarithmic scale.

Figure 17 shows the transfer characteristics of an IGZO/SiOC thin film transistor with SiOC heat treated at 200 °C as the gate insulator. There is a clear distinction between the bidirectional and unidirectional transfer characteristics. The width of the threshold voltage shift for ensuring the stability of the unidirectional transfer characteristics at V_DS_ ≥ 1 V is small. In addition, the bidirectional transfer characteristics for V_DS_ < 1 V are excellent.

**Figure 18:**
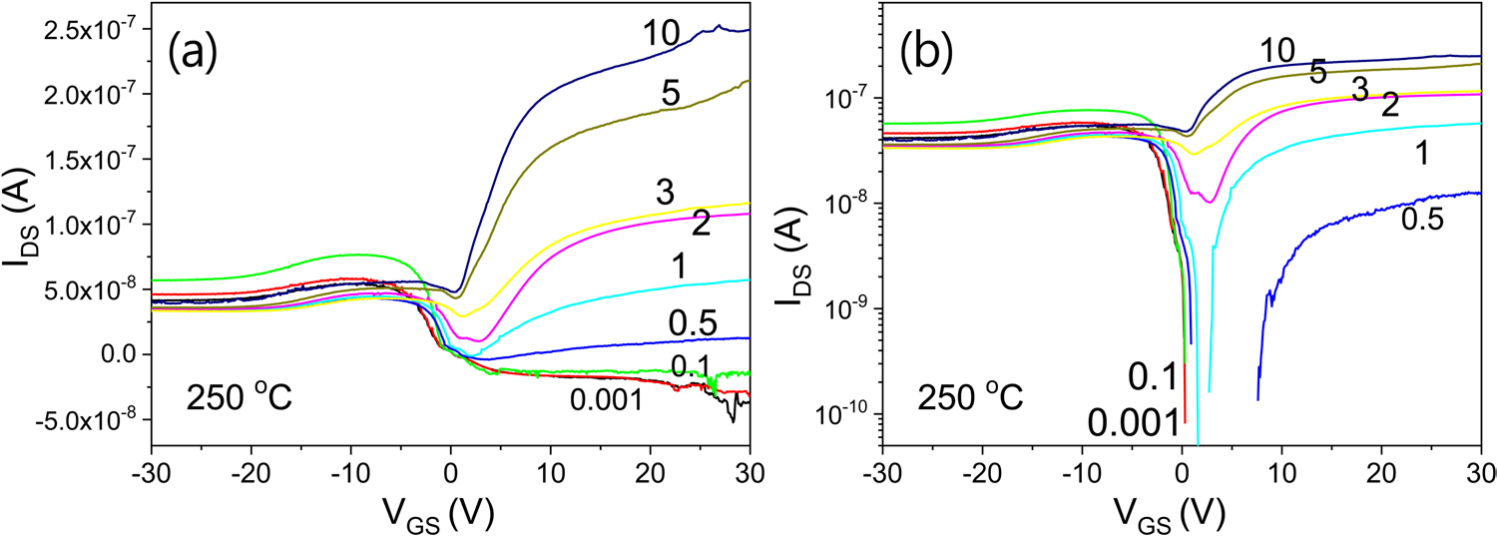
Transfer characteristics of an IGZO/SiOC thin film transistor with SiOC heat treated at 250 °C as the gate insulator, (**a**) on a linear scale and (**b**) on a logarithmic scale.

Figure 18 shows the transfer characteristics of an IGZO/SiOC thin film transistor with SiOC heat treated at 250 °C as the gate insulating film. It exhibits bidirectional transmission characteristics at V_DS_ < 1 V and unidirectional transmission characteristics at V_DS_ > 1 V.

**Figure 19:**
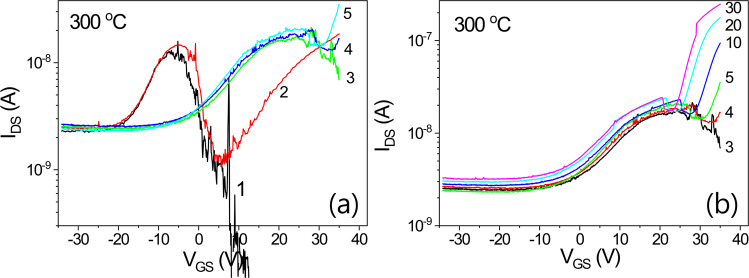
Transfer characteristics of an IGZO/SiOC thin film transistor with SiOC heat treated at 300 °C as the gate insulator, (**a**) transfer characteristics at V_DS_ ≤ 3 V and (**b**) transfer characteristics at V_DS_ > 3 V.

The transfer characteristics of an IGZO/SiOC thin film transistor with SiOC heat treated at 300 °C as the gate insulator are presented in Fig. 19. No bidirectional transmission characteristics are evident. Only bidirectional transfer characteristics are observed in the range of V_DS_ ≥ 1 V with increasing V_DS_.

**Figure 20:**
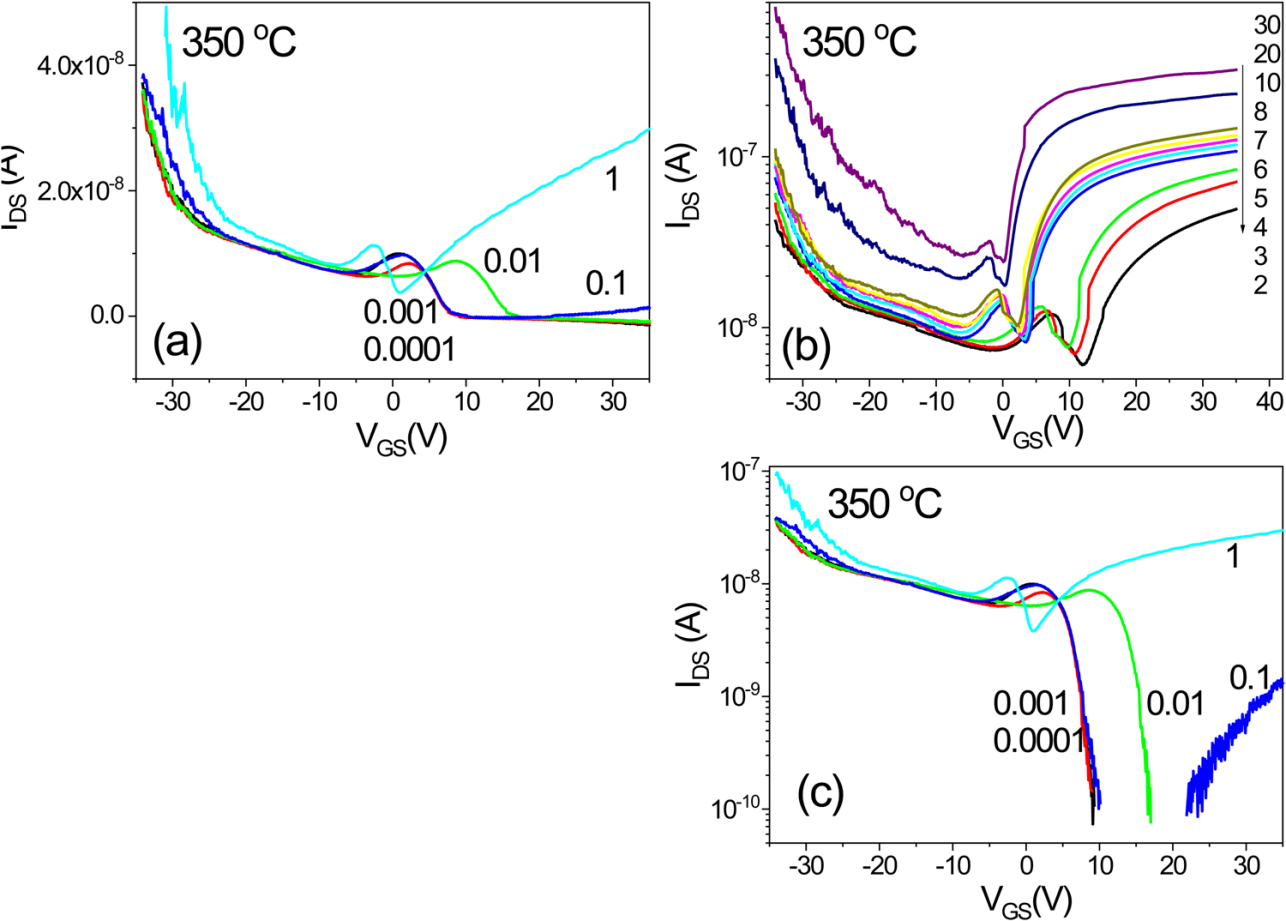
Transfer characteristics of an IGZO/SiOC thin film transistor with SiOC heat treated at 350 °C as the gate insulator, (**a**) transfer characteristics at V_DS_ ≤ 1 V, (**b**) transfer characteristics at V_DS_ > 1 V, and (**c**) the transfer characteristics at V_DS_ ≤ 1 V on a logarithmic scale.

Figure 20 shows the transfer characteristics of an IGZO/SiOC thin film transistor with SiOC heat treated at 350 °C as the gate insulator. In the rgnat of V_DS_ < 1 V, unstable bidirectional transmission characteristics are obserbed. However, in the area of V_DS_ > 1 V the unidirectional transmission characteristics are stable, and the threshold voltage shift phenomenon is observed.

**Figure 21:**
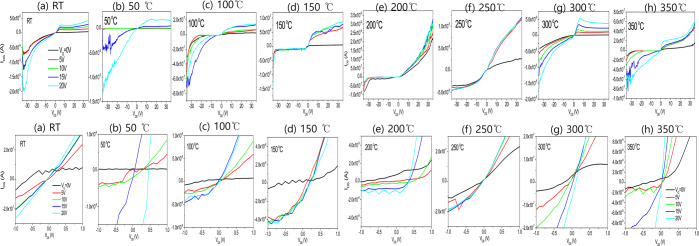
I_DS_-V_DS_ characteristics of transistors prepared with different heat treatment temperature.

Figure 21 shows the I_DS_-V_DS_ characteristic curve of the transistors prepared at different heat treatment temperatures. A Schottky contact was well formed in the sample treated at 200 °C.

**Figure 22:**
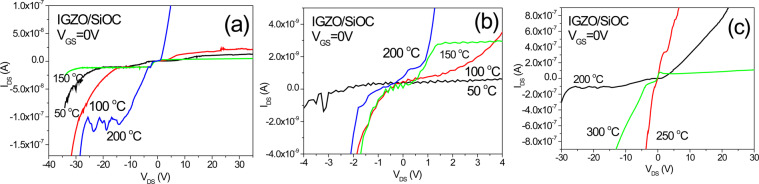
I_DS_-V_DS_ characteristic curves corresponding to differert heat treatment temperature at V_GS_ = 0 V; (**a**) sample heat treated at 200 °C with a Schottky contact near −20 V, (**b**) Schottky contact characteristics corresponding to different heat treatment temperature near 0 V, (**c**) samples heat treated at a temperature of 200 °C or greater.

Figure 22 shows at the I_DS_-V_DS_ characteristic curves corresponding to different heat treatment temperatures at V_GS_ = 0 V. Schottky contacts are found near −20 V and 0 V in the sample heat treated at 200 °C as shown in Fig. 22(a). Even in the range of −4 V < V_DS_ < 4 V, the Schottky contact is well formed in the 200 °C heat-treated sample. Figure 22(c) shows that it is difficult to form a Schottky contact with heat-treatment at greater than 200 °C.

**Figure 23:**
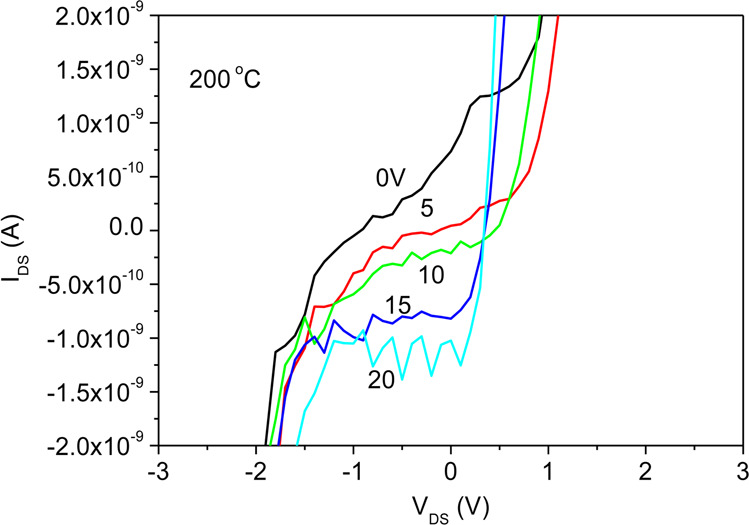
I_DS_-V_DS_ characteristic curves for V_GS_ = 0, 5, 10, 15, and 20 V of the sample treated at 200 °C.

Figure 23 illustrates the I_DS_-V_DS_ characteristic curves for V_GS_ values of 0, 5, 10, 15, and 20 V. The current I_DS_ decreases as the voltage V_GS_ increases. Despite an increase in either temperature or external voltage, a decrease in current is still observed due to the quantum anomalous Hall effect, and the devices exhibit magnetoresistance characteristics, meaning that current flows without a magnetic field being applied due to the large magnetic energy of the barrier potential of the phase insulator, which results in the highest current I_DS_ in the transistor with the SiOC film heat treated at 200 °C due to quantum tunneling in the phase isolator as shown in Fig. 11.

## Conclusion

Transistors prepared using only phase isolators without channels show bidirectional transmission characteristics due to the formation of spin currents induced by the barrier potential of the gate insulating film and the quantum spin Hall effect. By contrast, IGZO/SiOC transistors with IGZO channels displayed N-type unidirectional transmission characteristics, where the direction depends on the type of impurities introduced, as a result of the channel effect. A phase insulator presents a potential barrier and has a negative value of magnetic resistance; therefore, as the temperature increases the current decreases because the barrier potential arising due to recombination of the electron-hole pair (EHPs) becomes greater. Phase-isolator transistors do not show either N-typye or P-type transmission characteristics because they do not contain impurities; instead, they showed bidirectional transmission characteristics with a positive current I_DS_ flowing at a negative voltageV_GS_ due to the spin current and a negative I_DS_ flowing at a positive V_GS_.

## References

[1] Wang Y-ping (2018). High-temperature Dirac half-metal PdCl_3_:a promising candidate for realizing quantum anomalous Hall effect. J.Mater. Chem. C.

[2] Tsu R, Esaki L (1973). Tunneling in a finite superlattice. Appl. Phys. Lett..

[3] M-han Zhang, C-wen Zhang, P-ji Wang & S-shi Li, Prediction of high-temperature Chern insulator with half-metallic edge states in asymmetryfunctionalized stanene, *Nanoscale*, 2022, **10**, (2018). 10.1039/c8nr07503d 30357221

[4] Suzuki Y, Kubota H (2008). Spin-Torque Diode Effect and Its Application. J. Phys. Soc. Jpn..

[5] Oh T (2006). Origin of the SiCh_3_ Peak Position Shift in SiOC Films. Jpn. J. Appl. Phys..

[6] Jeffrey CK (2013). Evidence of ultra-low-k dielectric material degradation and nanostructure alteration of the Cu/ultra-low-k interconnects in time-dependent dielectric breakdown failure. Appl. Phys. Lett..

[7] Oh T (2015). Tunneling Phenomenon of Amorphous Indium-Gallium-Zinc-Oxide Thin Film Transistors for Flexible Display. Electronic Materials Lett..

[8] Oh T (2016). Tunneling Condition at High Schottky Barrier and Ambipolar Transfer Characteristics in Zinc Oxide Semiconductor Thin Film Transistor. Materials Research Bulletin.

[9] Oh T (2017). Relationship Between the Mobility and the Schottky Contact in Indium-Gallium-Zinc-Oxide Thin Film. Journal of nanoscience and nanotechnology.

[10] Oh T (2019). Correlation Between Currents, X-ray Diffraction Patterns and Transfer Characteristics of SnO_2_ Thin Film Transistor. Journal of nanoscience and nanotechnology.

[11] Oh T (2006). Organic Thin Film Transistor Using Pentacene and SiOC Film. IEEE Transactions on Nanotechnology.

[12] Barbagiovanni EG, Lockwood DJ, Simpson PJ, Goncharova LV (2014). Quantum confinement in Si and Ge nanostructures: Theory and experiment. Appl. Phys. Rev..

[13] Edvinsson T (2018). Optical quantum confinement and photocatalytic properties in two-, one- and zero-dimensional nanostructures. Royal society open science.

[14] Veldhorst M, Molenaar CG, Wang XL, Hilgenkamp H, Brinkman A (2012). Experimental realization of superconducting quantum interference devices with topological insulator junctions. Appl. Phys. Lett..

[15] Sodha MS, Dixit A, Srivastava S (2009). Photoelectric charging of dust particles: Effect of spontaneous and light induced field emission of electrons. Appl. Phys. Lett..

[16] Wiegmann PB (1978). One-dimensional Fermi system and plane xy model. J. Phys. C: Solid State Phys..

[17] Yang D (2013). A Large Magnetoresistance Effect in p–n Junction Devices by the Space-Charge Effect. Adv. Funct. Mater..

[18] Bagwell PF (1992). Suppression of the Josephson current through a narrow, mesoscopic, semiconductor channel by a single impurity. Phys Rev. B.

[19] Wang R, Erten O, Wang B, Xing DY (2019). Prediction of a topological p + ip excitonic insulator with parity anomaly. Nature Communications.

[20] Gray PV (1969). The Silicon-Silicon Dioxide. Proceedings of the IEEE..

[21] Chang LL, Esaki L, Tsu R (1974). Resonant tunneling in semiconductor double barriers. Appl. Phys. Lett..

[22] Tung RT (2014). The physics and chemistry of the Schottky barrier height. Appl. Phys. Rev..

[23] Delmo MP, Yamamoto S, Kasai S, Ono T, Kobayashi K (2009). Large positive magnetoresistive effect in silicon induced by the space-charge effect. Nature.

[24] Maserjian J, Zamani N (1982). Behavior of the Si/SiO_2_ interface observed by Fowler Nordheim tunneling. Appl Phys Lett..

[25] Volkov NV (2013). Extremely large magnetoresistance induced by optical irradiation in the Fe/SiO_2_/p-Si hybrid structure with Schottky barrier,. J. Appl. Phys..

[26] Schroeder H (2015). Poole-Frenkel effect as dominating current mechanism in thin oxide films An illusion?!. J. Appl. Phys..

[27] Mosbacker HL (2015). Role of near-surface states in Ohmic-Schottky conversion of Au contacts to ZnO. Appl. Phys. Lett..

[28] Frenkel J (1938). On pre-breakdown phenomena in insulators and electronic semi-conductors. Phys. Rev..

[29] Simmon JG (1967). Poole-Frenkel Effect and Schottky Effect in Metal-Insulator-Metal Systems. Phys. Rev..

[30] Oh T (2015). Electrical properties of nanoscale ZnS thin film transistor. Journal of Nanomaterials.

